# Hyperuricemia and diabetes mellitus when occurred together have higher risks than alone on all-cause mortality and end-stage renal disease in patients with chronic kidney disease

**DOI:** 10.1186/s12882-022-02755-1

**Published:** 2022-04-22

**Authors:** Cheng-Hung Li, Chia-Lin Lee, Yu-Cheng Hsieh, Cheng-Hsu Chen, Ming-Ju Wu, Shang-Feng Tsai

**Affiliations:** 1 Department of cardiovascular disease, Taichung Veterans General Hospital, Taichung, Taiwan; 2 Institute of Clinical Medicine, and Cardiovascular Research Center, National Yang-Ming University, Taipei, Taiwan; 3 Division of Endocrinology and Metabolism, Department of Internal Medicine, Taichung Veterans General Hospital, Taichung, Taiwan; 4 Department of Medical Research, Taichung Veterans General Hospital, Taichung, Taiwan; 5 Department of Public Health, College of Public Health, China Medical University, Taichung, Taiwan; 6 School of Medicine, National Yang-Ming University, Taipei, Taiwan; 7 Department of Post-Baccalaureate Medicine, College of Medicine, National Chung Hsing University, Taichung, Taiwan; 8 Division of Nephrology, Department of Internal Medicine, Taichung Veterans General Hospital, 160, Sec. 3, Taiwan Boulevard, Taichung, 407 Taiwan; 9 Department of Life Science, Tunghai University, Taichung, Taiwan

**Keywords:** Hyperuricemia, Diabetes mellitus, dialysis, Mortality, Chronic kidney disease

## Abstract

**Introduction:**

Hyperuricemia and diabetes mellitus (DM) are associated with increased mortality risk in patients with chronic kidney disease (CKD). Here we aimed to evaluate the independent and joint risks of these two conditions on mortality and end stage kidney disease (ESKD) in CKD-patients.

**Methods:**

This retrospective cohort study enrolled 4380 outpatients (with CKD stage 3–5) with mortality and ESKD linkage during a 7-year period (from 2007 to 2013). All-causes mortality and ESKD risks were analyzed by multivariable-adjusted Cox proportional hazards models (adjusted for age, sex, smoke, previous coronary arterial disease, blood pressure, and medications for hyperlipidemia, hyperuricemia and renin–angiotensin system inhibitors).

**Results:**

Overall, 40.5% of participants had DM and 66.4% had hyperuricemia. In total, 356 deaths and 932 ESKD events occurred during the 7 years follow-up. With the multivariate analysis, increased risks for all-cause mortality were: hyperuricemia alone, HR = 1.48 (1–2.19); DM alone, and HR = 1.52 (1.02–2.46); DM and hyperuricemia together, HR = 2.12 (1.41–3.19). Similar risks for ESKD were: hyperuricemia alone, HR = 1.34 (1.03–1.73); DM alone, HR = 1.59 (1.15–2.2); DM and hyperuricemia together, HR = 2.46 (1.87–3.22).

**Conclusions:**

DM and hyperuricemia are strongly associated with higher all-cause mortality and ESKD risk in patients with CKD stage 3–5. Hyperuricemia is similar to DM in terms of risk for all-cause mortality and ESKD. DM and hyperuricemia when occurred together further increase both risks of all-cause mortality and ESKD.

**Supplementary Information:**

The online version contains supplementary material available at 10.1186/s12882-022-02755-1.

## Introduction

Chronic kidney disease (CKD) is a public health burden worldwide due to its rapidly expanding patient populations, high risk of progression into end-stage kidney disease (ESKD), and poor prognosis of morbidity and mortality [1, 2]. The leading mortality of these patients is cardiovascular (CV) related deaths. With CKD progression, the CV outcomes become worse, including CV death, re-infarction, congestive heart failure, stroke, and resuscitation [3]. The most common cause of CKD is diabetes mellitus (DM) [4]. The 2002 National Cholesterol Education Program report designated DM a coronary heart disease risk equivalent, and DM is placed in the highest risk category [5]. Furthermore, DM and CKD are both potent independent risk factors for CV events and progression to ESKD [6, 7]. DM has huge burden of atherosclerosis related intimal thickening and CKD also causes medial calcification [8]. Therefore, patients with both conditions at the same time are therefore at exceedingly high risk of adverse events and would end with poor patient outcome.

The serum level of uric acid (UA) is also a risk factor for kidney disease [9], cardiovascular disease (CVD) [10–12], and atherosclerosis [13]. Serum UA is an independent risk factor for CKD, even in those without diabetes [14, 15]. Two large epidemiologic studies showed that UA is a major predictor for the incidence of renal disease [15, 16]. Moreover, hyperuricemia is often prevalent in CKD patients, and that is associated with a higher incidence of ESKD [16]. A number of studies showed that UA independently predicts the development of type 2 DM [17–19] and the progression of CKD [20]. For about 20 years, UA is known to be a potential risk factor for CKD and CVD with pathological implications [21, 22, 23]. Given the complex interplay among hyperuricemia, DM and the progression of CKD, we are interested to explore the complicated interactions regarding renal and patients outcomes. Here, we aimed to investigate the effects of DM and hyperuricemia on patient mortality and the development of ESKD in a large cohort of CKD patients.

## Methods

### Study cohort and definition

In this retrospective cohort study, we enrolled 4380 patients with CKD from the outpatients clinic of the nephrology department, Taichung Veterans General hospital (TVGH), Taiwan. Our hospital, a medical center with 1500 beds, is the referral hospital for the critically ill and difficult cases in central Taiwan. During the past 30 years, the CKD care program treated > 10,000 outpatients with CKD. CKD was defined as estimated glomerular filtration rate (eGFR) < 60 ml/min/1.73m^2^ for > 3 months irrespective of the cause. The eGFR equation was from Modification of Diet in Renal Disease (ml/min/1.732m^2^) [24]. DM was confirmed according the diagnosis of medical records. Hyperuricemia was defined as UA levels > 7.0 mg/dl for men, or > 6.0 mg/dl for women [21, 25]. These laboratory data were measured in our institute (TVGH).

### Data collections

We enrolled patients (> 20 years old) with CKD 3–5 from 2007 to 2013 in this study. After follow-up (2.5 years of mean duration) (end data of this study: 31-December-2015), the outcomes were analyzed by mortality and participants received regular dialysis at least 3 months or renal transplantation (ESKD). Their baseline variables were collected from medical records, including age, gender, stages of CKD, systolic blood pressure (SBP) (baseline and 1 year mean value), and diastolic blood pressure (DBP) (baseline and 1 year mean value), history of coronary artery disease, history of ever smoker, UA (baseline and 1 year mean value), baseline total cholesterol, usage of statin and usage of angiotensin-converting enzyme inhibitor (ACEi) or angiotensin II receptor blocker (ARB). The stage of CKD was based on the baseline renal function (the first laboratory data during the recruitment period of time). We chose the Modification of Diet in Renal Disease (MDRD) formula, instead of the Cockcroft and Gault formula, due to its superior accuracy in diabetic patients with impaired renal functions [26]. Although CKD-EPI (Epidemiology Collaboration) is more accurate than the MDRD equation for subjects with eGFR > 60 ml/min/1.73 m^2^, the MDRD formula is the one applied in the Taiwan National Database to evaluate dialysis initiation and CKD prevalence [27–29].

### Ethical approval and consent to participate

The study was approved by the institutional review board of the Taichung Veterans General Hospital approved the study (IRB TCVGH No: CE16235A-3) and all methods were carried out in accordance with relevant guidelines and regulations. The Informed consent was waived by the above ethics committee due to retrospective nature of the study.

### Statistical analysis

Data were presented as the mean ± standard deviation for continuous variables and proportions for categorical variables. An independent two-tailed *t* test was used for the comparison of continuous variables, and the differences between nominal variables were compared with the *Chi*-square test. Cox proportional hazards model was used to compare the hazard ratios (HRs) of all-cause mortality and dialysis event (adjusted for age, sex, ever smoke, CKD stage, 1 year mean SBP, use of statin, hyperuricemia drug usage, and ACEi/ARB usage.). Joint effects of hyperuricemia and DM on all-cause mortality and dialysis were also evaluated by Cox proportional hazards model, with adjusted for important covariates known to be associated with the predictors and outcomes of interests (adjusted for age, sex, ever smoke, CKD stage, 1 year mean SBP, use of statin, hyperuricemia drug usage, and ACEi/ARB usage.). In addition, since mortality was a competing event with dialysis, an extended Cox proportional hazards model was used to calculate the subdistribution hazard ratio (SHR) of dialysis as a sensitivity test [30]. We also analzyed the effect of DM and hyperuricemia on all-cause mortality and ESKD according to different stages of CKD. Statistical significance was set at *p* <  0.05. Statistical analyses were all carried out by using SPSS 22.0 (SPCC, Chicago, Illinois). Extended Cox proportional hazards model was analyzed by SAS software (version 9.4; SAS Institute, Inc., Cary, NC, USA).

## Results

### Baseline characteristics

Of the 4380 CKD patients, their median follow-up duration was 7 years. Among them, 40.5% had DM and 66.4% had hyperuricemia. Their mean age was 71 years and 63% were men. Baseline clinical and characteristics of patients are shown in Table 1 for the population, and in subpopulations according to DM or hyperuricemia. Of them, the mean age was 71 ± 14.8 years old. Patients with CKD were mostly in the stage 3 (47.4%). Mean SBP and DPP were 134 ± 18.7 and 75 ± 11.1 mmHg. Baseline and one-year later UA were both around 8 mg/dl (8.1 ± 2.5 and 8.0 ± 2.1 mg/dl, respectively). In DM related CKD patients with hyperuricemia were younger (71 ± 12.2 vs. 73 ± 18.8, *p* = 0.001), of later stages of CKD (*p* <  0.001), more frequent smokers (43.4% vs. 35.4%, *p* = 0.001), higher baseline SBP (138 ± 19.5 vs. 135 ± 18.1 mmHg, *p* = 0.013) and DBP (75 ± 10.8 vs. 74 ± 10.3 mmHg, *p* = 0.043), and higher one-year-later mean UA (9.1 ± 2.1 vs. 5.9 ± 0.9 mg/dl, *p* <  0.001). For CKD patients without DM, those with hyperuricemia were younger (70 ± 16.8 vs. 72 ± 15.3, *p* <  0.001), more male gender (65.7 vs. 59.1%, *p* <  0.001), of later stages of CKD (*p* <  0.001), more frequent smokers (38.6 vs. 34.1%, *p* = 0.027), higher one-year-later mean UA (8.9 ± 1.5 vs. 5.9 ± 0.9 mg/dl, *p* <  0.001), and more with ACEi/ARB usage (52.7% vs. 48.6%, *p* = 0.045).

**Table 1:** Basic characteristics stratified by DM and uric acid level

		DM			No DM		
Variable	Overall (*N* = 4380)	UA > 7 (*n* = 1174)	UA < 7 (*n* = 602)	*p*-value	UA > 7 (*n* = 1737)	UA < 7 (*n* = 867)	*p*-value
Age (y)	71 ± 14.8	71 ± 12.2	73 ± 11.8	0.001^a^	70 ± 16.8	72 ± 15.3	< 0.001^a^
Male sex	2747(62.7)	741(63.1)	352(58.5)	0.057^b^	1142(65.7)	512(59.1)	< 0.001^b^
CKD stage				< 0.001^b^			< 0.001^b^
3	2076(47.4)	461(39.3)	323(53.7)		803(46.2)	489(56.4)	
4	1364(31.1)	414(35.3)	200(33.2)		530(30.5)	220(25.4)	
5	940(21.5)	299(25.5)	79(13.1)		404(23.3)	158(18.2)	
Ever smoke	1688(38.5)	509(43.4)	213(35.4)	0.001^b^	670(38.6)	296(34.1)	0.027^b^
Previous CAD	225(5.1)	86(7.3)	31(5.1)	0.08^b^	69(4.0)	39(4.5)	0.526^b^
Baseline SBP (mmHg)	134 ± 18.7	138 ± 19.5	135 ± 18.1	0.013^a^	133 ± 18.5	132 ± 17.9	0.40^a^
1 year mean SBP (mmHg)	134 ± 16.1	137 ± 16.4	135 ± 15.6	0.014^a^	133 ± 16.1	131 ± 15.3	0.118^a^
Baseline DBP (mmHg)	75 ± 11.1	75 ± 10.8	74 ± 10.3	0.043^a^	75 ± 11.4	75 ± 11.2	0.34^a^
1 year mean DBP (mmHg)	74 ± 9.5	75 ± 8.9	74 ± 8.9	0.116^a^	75 ± 10.0	74 ± 9.4	0.092^a^
Baseline UA (mg/dl)	8.1 ± 2.5	9.2 ± 3.1	6.0 ± 1.1	< 0.001^a^	9.1 ± 1.8	6.0 ± 1.1	< 0.001^a^
1 year mean UA (mg/dl)	8.0 ± 2.1	9.1 ± 2.1	5.9 ± 0.9	< 0.001^a^	8.9 ± 1.5	5.9 ± 0.9	< 0.001^a^
Baseline cholesterol (mmol/l)	185.2 ± 51.4	183.5 ± 53.0	180.1 ± 50.8	0.214^a^	187.9 ± 50.1	185.7 ± 51.9	0.325^a^
Statin usage	1522(34.7)	544(46.3)	279(46.3)	0.997^b^	473(27.2)	226(26.1)	0.528^b^
ACEi/ARB usage	2540(58.0)	802(68.3)	401(66.6)	0.468^b^	916(52.7)	421(48.6)	0.045^b^
Uric acid-lowering drugs	1228 (28.0)	306 (26.1)	107 (17.8)	< 0.0001	599 (34.5)	216 (24.9)	< 0.0001

Continuous variables are expressed as mean ± SD

Categorical data are presented as numbers (percentages). ^a^Independent T-Test. ^b^Chi-Square Test

### Association between hyperuricemia and all-cause mortality and ESKD

During the follow-up periods, 356 (8.1%) deaths occurred and 932 (21.3%) participants received regular dialysis at least 3 months (ESKD). Hazard ratios (HRs) with 95% confidence intervals (CIs) for each combination of predictors are summarized in Table 2. For all-cause mortality, the univariate analysis showed the following associated factors: DM (HR = 1.49, 95%CI = 1.21–1.83)(*p* = 0.0002), hyperuricemia (HR = 1.39, 95%CI = 1.1–1.75)(*p* = 0.0058), older age (HR = 1.06, 95%CI = 1.05–1.07)(*p* <  0.0001), male gender (HR = 1.46, 95%CI = 1.16–1.84)(*p* = 0.0012), ever smoker (HR = 1.74, 95%CI = 1.41–2.14) (*p* <  0.0001), CKD stage 4 compared to stage 3 (HR = 1.61, 95%CI = 1.27–2.05)(*p* <  0.0001), stage 5 compared to stage 3 (HR = 1.56, 95%CI = 1.19–2.05)(*p* = 0.0015), medication for hyperuricemia (HR = 1.33, 95%CI = 1.07–1.65) (*p* = 0.0105), and statin (HR = 0.71, 95%CI = 0.56–0.9)(*p* = 0.004). Further multivariate analysis for all-cause mortality still showed the following associated factors: DM (HR = 1.46, 95%CI = 1.13–1.88) (*p* = 0.004), hyperuricemia (HR = 1.44, 95%CI = 1.09–1.91)(*p* = 0.0114), older age (HR = 1.05, 95%CI = 1.04–1.06) (*p* < 0.0001), ever smoker (HR = 1.58, 95%CI = 1.16–2.16) (*p* = 0.0042), CKD stage 4 compared to stage 3 (HR = 1.88, 95%CI = 1.42–2.49) (*p* < 0.0001), CKD stage 5 compared to stage 3(HR = 1.85, 95%CI = 1.3–2.63) (*p* = 0.0007), and statin (HR = 0.67, 95%CI = 0.5–0.9) (*p* = 0.0085).

**Table 2:** Hazard ratios (HRs) (95% CI) on all-cause mortality and ESKD (univariate and multivariate analysis)

	Univariate analysis				Multivariate analysis			
	Mortality	*p* value	ESKD	*p* value	Mortality	*p* value	ESKD	*p* value
DM at least	1.49(1.21–1.83)	0.0002	1.49(1.31–1.7)	< 0.0001	1.46(1.13–1.88)	0.004	1.77(1.49–2.10)	< 0.0001
Hyperuricemia at least	1.39(1.1–1.75)	0.0058	1.71(1.48–1.99)	< 0.0001	1.44(1.09–1.91)	0.0114	1.43(1.19–1.73)	0.0002
Age	1.06(1.05–1.07)	< 0.0001	0.97(0.97–0.98)	< 0.0001	1.05(1.04–1.06)	< 0.0001	0.97(0.97–0.98)	< 0.0001
Gender (male v.s. female)	1.46(1.16–1.84)	0.0012	0.65(0.58–0.74)	< 0.0001	0.96(0.67–1.37)	0.8161	1.01(0.82–1.24)	0.9394
Ever smoker (yes v.s. no)	1.74(1.41–2.14)	< 0.0001	0.9(0.78–1.03)	0.1124	1.58(1.16–2.16)	0.0042	1.16(0.95–1.43)	0.1537
CKD stage (stage4 v.s. stage3)	1.61(1.27–2.05)	< 0.0001	4.13(3.33–5.14)	< 0.0001	1.88(1.42–2.49)	< 0.0001	4.33(3.29–5.70)	< 0.0001
CKD stage (stage5 v.s. stage3)	1.56(1.19–2.05)	0.0015	22.43(18.29–27.52)	< 0.0001	1.85(1.30–2.63)	0.0007	24.16(18.59–31.38)	< 0.0001
Mean SBP (mmHg)	1(0.99–1.00)	0.3511	1.02(1.02–1.03)	< 0.0001	1(0.99–1.00)	0.2826	1.02(1.01–1.02)	< 0.0001
Medications for hyperuricemia (yes v.s. no)	1.33(1.07–1.65)	0.0105	0.93(0.80–1.07)	0.2845	1.2(0.92–1.56)	0.1828	0.84(0.70–1.02)	0.0746
Statin (yes v.s. no)	0.71(0.56–0.90)	0.004	1.08(0.94–1.24)	0.2621	0.67(0.50–0.90)	0.0085	1.18(0.99–1.40)	0.0646
ACEi or ARB (yes v.s. no)	1.02(0.82–1.26)	0.8836	1.03(0.91–1.18)	0.6346	1.13(0.87–1.47)	0.3488	0.94(0.80–1.12)	0.4984

Note: adjusted for age, sex, ever smoke, CKD stage, 1 year mean SBP, use of statin, hyperuricemia drug usage, ACEi/ARB usage

For ESKD, the univariate analyses showed the following associated factors: DM (HR = 1.49, 95%CI = 1.31–1.7)(*p* < 0.0001), hyperuricemia (HR = 1.71, 95%CI = 1.48–1.99) (*p* < 0.0001), younger age (HR = 0.97, 95%CI = 0.97–0.98) (*p* < 0.0001), male gender (HR = 0.65, 95%CI = 0.58–0.74) (*p* < 0.0001), CKD stage 4 compared to stage 3 (HR = 4.13, 95%CI = 3.33–5.14) (*p* < 0.0001), and stage 5 compared to stage 3 (HR = 22.43, 95%CI = 18.29–27.52) (*p* < 0.0001). Further multivariate analysis for ESKD still showed the following associated factors: DM (HR = 1.77, 95%CI = 1.49–2.1) (*p* < 0.0001), hyperuricemia (HR = 1.43, 95%CI = 1.19–1.73) (*p* = 0.0002), older age (HR = 0.97, 95%CI = 0.97–0.98) (*p* < 0.0001), CKD stage 4 compared to stage 3 (HR = 4.33, 95%CI = 3.29–5.7) (*p* < 0.0001), CKD stage 5 compared to stage 3(HR = 24.16, 95%CI = 18.59–31.38) (*p* < 0.0001), and mean SBP (HR = 1.02, 95%CI = 1.01–1.02) (*p* < 0.0001).

### Analyses of effects of DM and hyperuricemia on all-cause mortality, and ESKD

**Fig. 1:**
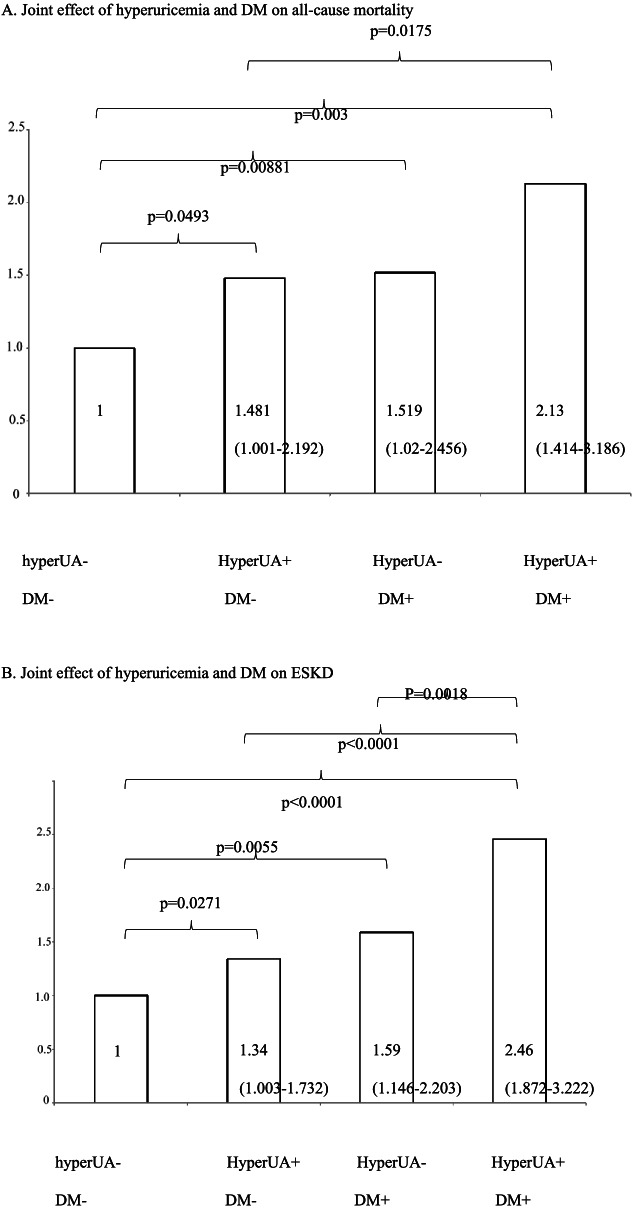
**A** The significance of HRs for mortality: DM and hyperuricemia together (HR = 2.13) > DM alone (HR = 1.52) = hyperuricemia alone (HR = 1.48) > neither DM nor hyperuricemia (HR = 1). **B** For ESKD, the significance of HRs: DM and hyperuricemia together (HR = 2.46) > DM alone (HR = 1.59) = hyperuricemia alone (HR = 1.34) > neither DM nor hyperuricemia (HR = 1)

Further analysis of DM, hyperuricemia and both DM and hyperuricemia on all-cause mortality, ESKD and CV death are shown in supplementary Table 1. Results of Cox proportional hazard models are shown in Table 3 (univariate and multivariate analyses). All were compared with the reference (neither DM nor hyperuricemia). In univariate analyses, hyperuricemia alone, DM alone, and DM and hyperuricemia together showed higher risk for all-cause mortality (HR = 1.46, 95%CI = 1.05–2.02 (*p* = 0.0228); HR = 1.59, 95%CI = 1.07–2.36 (*p* = 0.023); HR = 2.13, 95%CI = 1.53–2.98 (*p* < 0.0001)). The higher risks for all-cause mortality were still found in the multivariate analysis: hyperuricemia alone, HR = 1.48, 95%CI = 1.2–2.19 (*p* = 0.0493); DM alone, HR = 1.52, 95%CI = 1.02–2.46 (*p* = 0.0088); DM and hyperuricemia together, HR = 2.12, 95%CI = 1.41–3.19 (*p* = 0.0003). We found similar results with ESKD. The univariate analysis still showed the following associated factors: hyperuricemia alone, HR = 1.72, 95%CI = 1.40–2.10 (*p* < 0.0001); DM alone, HR = 1.48, 95%CI = 1.14–1.92 (*p* = 0.003); hyperuricemia and DM together, HR = 2.61, 95%CI = 2.11–3.22 (*p* = < 0.0001). The multivariate analysis revealed the following assocaited factors: hyperuricemia alone, HR = 1.34, 95%CI = 1.03–1.73 (*p* = 0.0271); DM alone, HR = 1.59, 95%CI = 1.15–2.2 (*p* = 0.0055); hyperuricemia and DM together, HR = 2.46, 95%CI = 1.87–3.22 (*p* < 0.0001). This joint effects of DM and hyperuricemia on ESKD and all-cause mortality are also shown in Fig. 1, including all-cause mortality (Fig. 1A) and ESKD (Fig. 1B). The Kaplan-Meier plot for all-cause mortality or ESKD were also showed similar results (Supplementary Fig. 1A for ESKD and Fig. 1B for mortality).

**Table 3:** Cox Proportional Hazards Models for patient all-cause mortality and ESKD divided by DM and hyperuricemia

	No. of Events	Univariate 95% CI	*p*-value	Multivariate 95% CI^a^	*p*-value
**All-cause mortality**					
Neither DM nor Hyperuricemia	49	REF.		REF.	–
Hyperuricemia only	142	1.46(1.05–2.02)	0.0228	1.48(1–2.19)	0.0493
DM only	48	1.59(1.07–2.36)	0.023	1.52(1.02–2.46)	0.0088
Both DM and Hyperuricemia	117	2.13(1.53–2.98)	<.0001	2.12(1.41–3.19)	0.0003
**ESKD**					
Neither DM nor Hyperuricemia	121	REF.		REF.	–
Hyperuricemia only	388	1.72 (1.40–2.10)	<.0001	1.34(1.03–1.73)	0.0271
DM only	107	1.48 (1.14–1.92)	0.003	1.59(1.15–2.2)	0.0055
Both DM and Hyperuricemia	316	2.61 (2.11–3.22)	<.0001	2.46(1.87–3.22)	<.0001
**Dialysis with considering competing risk of mortality**					
Neither DM nor Hyperuricemia	121	REF.		REF.	
Hyperuricemia only	388	1.671(1.366–2.044)	<.0001	1.266(0.97–1.651)	0.0825
DM only	107	1.428(1.106–1.844)	0.0064	1.529(1.064–2.197)	0.0218
Both DM and Hyperuricemia	316	2.467(2.004–3.036)	<.0001	2.251(1.702–2.978)	<.0001

Note: adjusted for age, sex, ever smoke, previous CAD, 1 year mean SBP, use of statin, hyperuricemia drug usage, ACEi/ARB usage

Cox proportional multivariate hazards ratio for patient all-cause mortality and ESKD divided by DM and hyperuricemia ind different stages of CKD (strage 3, 4, and 5) were shown in Supplementary Table 2 (all-cause mortality) and Table 3 (ESKD). As for all-cause mortality (Supplementary Table 2), we found the following assciated facotrs: hyperuricemia alone in CKD stage 4, HR = 2.32, 95%CI = 1.03–5.21 (*p* = 0.0418); DM alone in CKD stage 4, HR = 3.282, 95%CI = 1.35–7.97 (*p* = 0.0087); DM and hyperuricemia together in CKD stage 4, HR = 3.99, 95%CI = 1.77–8.99 (*p* = 0.0009); DM and hyperuricemia in CKD stage 5, HR = 3.105, 95%CI = 1.04–9.30 (*p* = 0.0429). As for ESKD (Supplementary Table 3), we found the following assciated facotrs: DM alone in CKD stage 3, HR = 3.061, 95%CI = 1.235–7.586 (*p* = 0.0157); DM and hyperuricmie together in CKD stage 3, HR = 3.556, 95%CI = 1.616–7.824 (*p* = 0.0016); DM alone in CKD stage 4, HR = 2.396, 95%CI = 1.310–4.383 (*p* = 0.0046); DM and hyperuricemia together in CKD stage 4, HR = 2.862, 95%CI = 1.668–4.910 (*p* = 0.0001); DM and hyperuricemia together in CKD stage 5, HR = 1.815, 95%CI = 1.296–2.541 (*p* = 0.0005).

## Discussion

The principal finding of this study is that the conditions hyperuricemia and DM when occurred together further increased risk of ESKD and all-cause mortality compared with the conditions existed alone. Results were independent of traditional risk factors such as age, gender, BP, and smoking in patients with CKD. Results are consistent with previous studies [15, 31, 32] showing that hyperuricemia is an independent risk factor for ESKD in the general population and in patients with CKD [33–36]. The higher risk on all-cause mortality and ESKD remained significant after adjustment for multiple confounding factors. However, other epidemiologic studies revealed uncertain conclusion because of differences in methodologies and impact on serum UA concentrations by even subtle changes in kidney function in the general population [37]. The causal role of serum UA in kidney disease, hypertension, or DM remains debatable regarding the general population [37]. For patients of CKD stage 3 to 5, we reported earlier in a retrospective study that hyperuricemia is associated with higher risk of incident renal replacement therapy and all-cause mortality [33]. The potential mechanisms that hyperuricemia contributes to CKD progression include a poorer renal perfusion via stimulation of afferent arteriolar vascular smooth muscle cell proliferation [16, 38–40]. Hyperuricemia may lead to acute UA nephropathy [41], chronic urate nephropathy [42], gout related renal injury and anesthesia related nephropathy. Many conditions associated with hyperuricemia in CKD patients could also contribute the progression of CKD.

To our knowledge, this is the first study to show that hyperuricemia is a risk equivalent to DM for all-cause mortality (HR = 1.48 vs. HR = 1.52) and ESKD (HR = 1.34 vs. HR = 1.59) in patients with CKD stage 3 to 5. Gout is a risk factor for CVD [43], CV mortality [44] and all-cause mortality [44, 45]. The possible mechanism is related to hyperuricemia [46]. Hyperuricemia in the absence of gout has a risk of stroke 1.47 times higher [47] and a risk of coronary heart disease 1.34 times higher [48]. From a retrospective study on claims database study, gout has a risk equivalent to DM for the incidence of stroke [46]. Hyperuricemia is linked to impaired production of nitric oxide [49, 50] the activation of renin-angiotensin system [51]. Both of the above factors cause endothelial dysfunction [52, 53], and further contribute to hypertension [54, 55] and hyperuricemia [56, 57]. Studies showed that UA stimulates the proliferation of vascular smooth muscle cells [58–60]. Hyperuricemia-related monosodium urate crystals [61, 62] may cause atherosclerosis with more coagulation [63]. Both hyperuricemia and DM are linked to CVD and all-cause mortality. In addition, hyperuricemia-related gout usually requires treatment with nonsteroidal anti-inflammatory drug (NSAID). NSAID is also associated with higher CV mortality and all-cause mortality [64–66]. Finally, the hyperuricemia may be related to the use of diuretics, a medication which is typically used in patients with heart failure with pulmonary edema and unstable heart function. Both low cardiac output and diuretic therapy reduce UA excretion. Hence hyperuricemia is likely a good maker for poor heart function and higher risk for mortality [67]. In summary, regarding all-cause mortality, hyperuricemia has a risk equivalent to DM.

Hyperuricemia was also a risk equivalent to DM for ESKD in this CKD cohort in Taiwan, which has the highest incidence of ESKD worldwide [68]. In addition to potential mechanisms that hyperuricemia contributes to CKD progression by reducing renal perfusion via stimulated proliferation of afferent arteriolar vascular smooth muscle cells [16, 38–40], and the over-use of NSAID for gout attack also threatened CKD progression. Once gout attack, patients got used to taking NSAIDs for pain relief even if definite evidence of renal toxicity of NSAIDs. From a Nationwide study in Taiwan (109,400 incident chronic ESKD patients from 1998 to 2009) [69], adjusted odds ratio (OR) was 2.73 (95% CI: 2.62–2.84) for nonselective NSAIDs and 2.17 (95% CI: 1.83–2.57) for celecoxib. Compared with the non-users, users of oral NSAID were 3.74 times more likely to develop dialysis-required ESKD. This severe renal risk could be even greater for people who had recently used the parenteral form of NSAIDs (adjusted OR: 8.66) [69]. About 30% dialytic patients still took NSAID 1 year before the initiation of dialysis (2018 Annual Report on Kidney Disease, Taiwan) [70]. Moreover, the number of patients taking NSAID was likely under-estimated because its over-the-counter availability in Taiwan.

Our patients with both DM and hyperuricemia had more increased risk of ESKD (HR = 2.46) than either DM or hyperuricemia alone. The joint effect was greater than the additive HR (1.59*1.34 = 2.13) of DM and hyperuricemia. The discrepancy may be due to synergistic or potentiating effects. First, DM and hyperuricemia share some similar mechanism for renal injury in patients with CKD, but other UA associated mechanisms for renal injury may be independent from DM (like acute UA nephropathy [41], chronic urate nephropathy [42], gout related renal injury and NSAID related nephropathy [69]). Such additional mechanisms of hyperuricemia and gout-related renal injury could lead to higher risk of ESKD in DM-related CKD. Therefore, even hyperuricemia and DM shared similar mechanisms for CKD progression, the combined risks of DM and hyperuricemia for ESKD appeared higher than DM or hyperuricemia alone. Second, there are several kinds of synergistic effects between DM and hyperuricemia on CKD progression. Initial hyperuricemia is an independent risk factor for the progression of diabetic kidney disease (DKD) [71]. High serum UA levels potentiate CKD progression in patients with type 2 DM [72]. Initially, activation of the renin-angiotensin system causes glomerular hyperfiltration [51, 73], a finding characterizes diabetic kidney disease (DKD) and CKD. Thus, hyperfiltration is potentiated under hyperuricemia in patients with both CKD and type 2 DM. In addition, UA stimulates proliferation of vascular smooth muscle cells and their oxidative stress [59], leading to progression of CKD. The oxidative stress and inflammation are typical findings of DKD [74, 75]. Furthermore, UA-related alleles of SLC2A9 rs11722228, SLC2A9 rs3775948, ABCG2 rs2231142 affect DKD susceptibility in the Chinese patients with type 2 DM [76]. A clinical study on 15-year follow-up supported the contribution of hyperuricemia on CKD progression [71]. In another clinical study, febuxostat preserves eGFR in patients of DKD, at levels beyond glycemic control [77]. Low-doses allopurinol reduce the severity of proteinuria in type 2 DM, probably through decreased serum UA [78]. Therefore, UA can be considered as a mediator of DKD [79] and the joint effect of hyperuricemia and DM on ESKD could be synergistic.

There are some limitations of our present study. First, causal effect of hyperuricemia on ESKD and all-cause mortality cannot be established. Second, we did not record the gout condition and the usage of NSAID. Third, our results cannot be generalized to patients covering all stages of CKD. Finally, for patients with CKD under critical status, the result cannot be generalized to this population, neither. Despite these limitations, DM and hyperuricemia having joint effect on ESKD and all-cause mortality remains a robust finding.

## Conclusions

Both DM and hyperuricemia are strongly associated with more all-cause mortality and ESKD risk in patients with CKD stage 3–5. In these patients, hyperuricemia has same effect as DM on risk of all-cause mortality and ESKD. Joint effects of DM and hyperuricemia further increase risk of all-cause mortality and ESKD.

## Supplementary Information

**Additional file 1.**

## Data Availability

All data generated or analysed during this study are included in this published article and its supplementary information files.

## Abbreviations

ACEi: Angiotensin- converting enzyme inhibitors
ARB: Angiotensin II receptor blockers
CI: Confidence interval
CKD: Chronic kidney disease
CKD-EPI: Chronic kidney disease-Epidemiology Collaboration
CV: Cardiovascular
DKD: Diabetic kidney disease
DM: Diabetes Mellitus
eGFR: Estimated glomerular filtration rate
ESKD: End-stage kidney disease
HR: Hazard ratio
MDRD: Modification diet of renal disease
NSAID: Nonsteroidal anti-inflammatory drug
UA: Uric acid
