# Supplementary material for: Hyperuricemia and diabetes mellitus when occurred together have higher risks than alone on all-cause mortality and end-stage renal disease in patients with chronic kidney disease
Source: BMC Nephrol. 2022 Apr 22;23:157. doi: 10.1186/s12882-022-02755-1 (PMC9034537; doi:10.1186/s12882-022-02755-1)

**Supplementary table 1.** Comparison of hazards rations for all-cause mortality, and ESKD between all situations of DM and hyperuricemia

| **All-cause mortality** | | | | |
| --- | --- | --- | --- | --- |
|  | HyperUA-, DM- | HyperUA+, DM- | HyerUA-, DM+ | HyperUA+, DM+ |
| HyperUA-, DM- | Reference | 1.481 (1.001-2.192),  p=0.0493 | 1.519 (1.02-2.456), p=0.0088 | 2.123 (1.414-3.186), p=0.0003 |
| HyperUA+, DM- |  | Reference | 1.025 (0.689-1.526), p=0.9016 | 1.433 (1.065-1.928), p=0.0175 |
| HyerUA-, DM+ |  |  | Reference | 1.397 (0.934-2.09), p=0.1033 |
| HyperUA+, DM+ |  |  |  | Reference |
| **ESKD** | | | | |
|  | HyperUA-, DM- | HyperUA+, DM- | HyerUA-, DM+ | HyperUA+, DM+ |
| HyperUA-, DM- | Reference | 1.337 (1.033-1.732),  p=0.0271 | 1.589 (1.146-2.203), p=0.0055 | 2.456 (1.872-3.222), p<0.0001 |
| HyperUA+, DM- |  | Reference | 1.188 (0.904-1.561), p=0.216 | 1.837 (1.509-2.236), p<0.0001 |
| HyerUA-, DM+ |  |  | Reference | 1.546 (1.176-2.033), p=0.0018 |
| HyperUA+, DM+ |  |  |  | Reference |

HyperUA: hyperuricemia; DM: diabetes mellitus

**Supplementary table 2. Cox Proportional Multivariate Hazards Models for all-cause mortality divided by DM and hyperuricemia ind different stages of CKD (strage 3, 4, and 5)**

|  | HR | 95% C.I. | Chi Square |
| --- | --- | --- | --- |
| CKD stage 3 | | | |
| Neither DM nor Hyperuricemia | Reference |  |  |
| Hyperuricemia only | 1.214 | 0.726-2.032 | 0.4601 |
| DM only | 1.016 | 0.526-1.961 | 0.9619 |
| Both DM and Hyperuricemia | 1.147 | 0.633-2.080 | 0.6508 |
| CKD stage 4 | | | |
| Neither DM nor Hyperuricemia | Reference |  |  |
| Hyperuricemia only | 2.318 | 1.032-5.21 | 0.0418 |
| DM only | 3.282 | 1.351-7.974 | 0.0087 |
| Both DM and Hyperuricemia | 3.985 | 1.766-8.991 | 0.0009 |
| CKD stage 5 | | | |
| Neither DM nor Hyperuricemia | Reference |  |  |
| Hyperuricemia only | 1.956 | 0.658-5.812 | 0.2274 |
| DM only | 1.070 | 0.191-5.980 | 0.9388 |
| Both DM and Hyperuricemia | 3.105 | 1.037-9.298 | 0.0429 |

*p-value <0.05. **adjusted for age, sex, ever smoke, previous CAD, 1 year mean SBP, use of statin, hyperuricemia drug usage, and ACEi/ARB usage.

**Supplementary table 3. Cox Proportional Multivariate Hazards Models for ESKD divided by DM and hyperuricemia ind different stages of CKD (strage 3, 4, and 5)**

| CKD stage 3 | | | |
| --- | --- | --- | --- |
| Neither DM nor Hyperuricemia | Reference |  |  |
| Hyperuricemia only | 1.42 | 0.628-3.207 | 0.3993 |
| DM only | 3.061 | 1.235-7.586 | 0.0157 |
| Both DM and Hyperuricemia | 3.556 | 1.616-7.824 | 0.0016 |
| CKD stage 4 | | | |
| Neither DM nor Hyperuricemia | Reference |  |  |
| Hyperuricemia only | 1.498 | 0.886-2.532 | 0.1313 |
| DM only | 2.396 | 1.310-4.383 | 0.0046 |
| Both DM and Hyperuricemia | 2.862 | 1.668-4.910 | 0.0001 |
| CKD stage 5 | | | |
| Neither DM nor Hyperuricemia | Reference |  |  |
| Hyperuricemia only | 1.152 | 0.845-1.569 | 0.3708 |
| DM only | 1.038 | 0.625-1.725 | 0.8857 |
| Both DM and Hyperuricemia | 1.815 | 1.296-2.541 | 0.0005 |

*p-value <0.05. **adjusted for age, sex, ever smoke, previous CAD, 1 year mean SBP, use of statin, hyperuricemia drug usage, and ACEi/ARB usage.

**Supplementary figure 1. Kaplan-Meier plot of ESKD and all-cause mortality.**

**Figure 1A. Kaplan-Meier plot for ESKD**


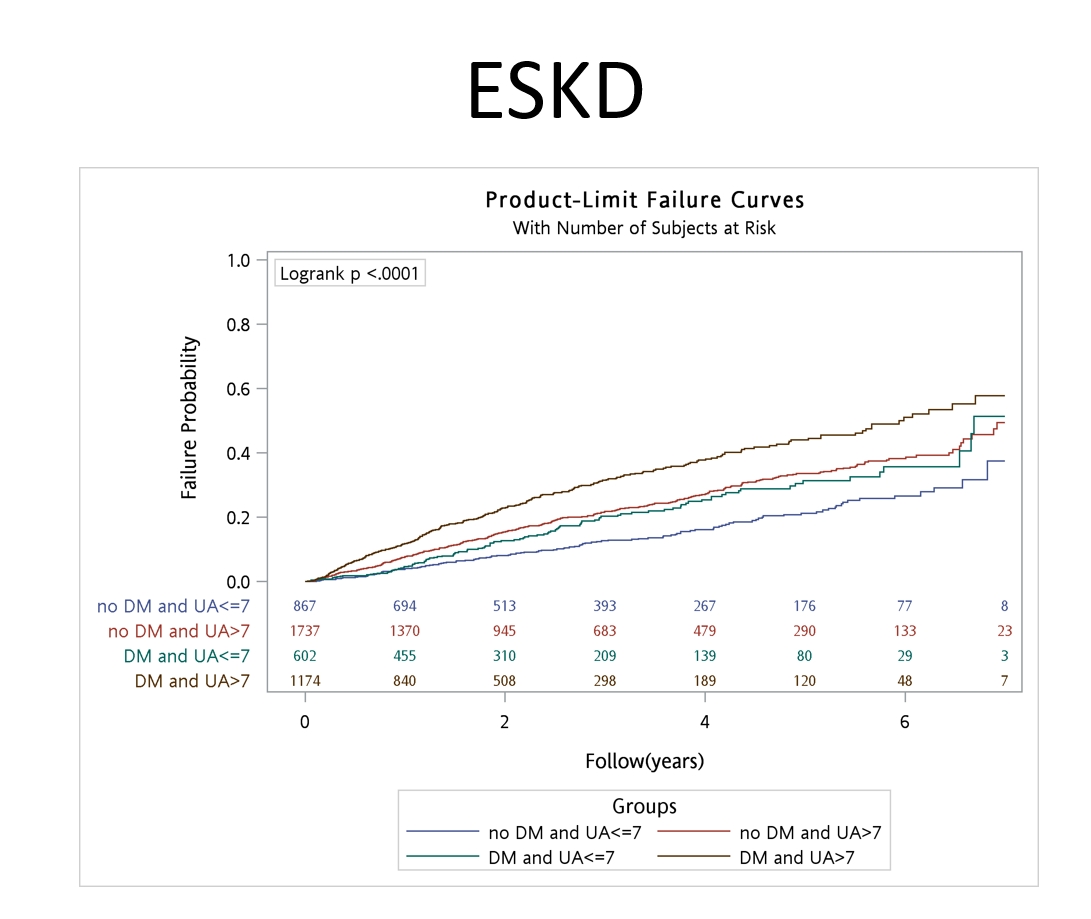


**Figure 1B. Kaplan-Meier plot for all-cause mortality**


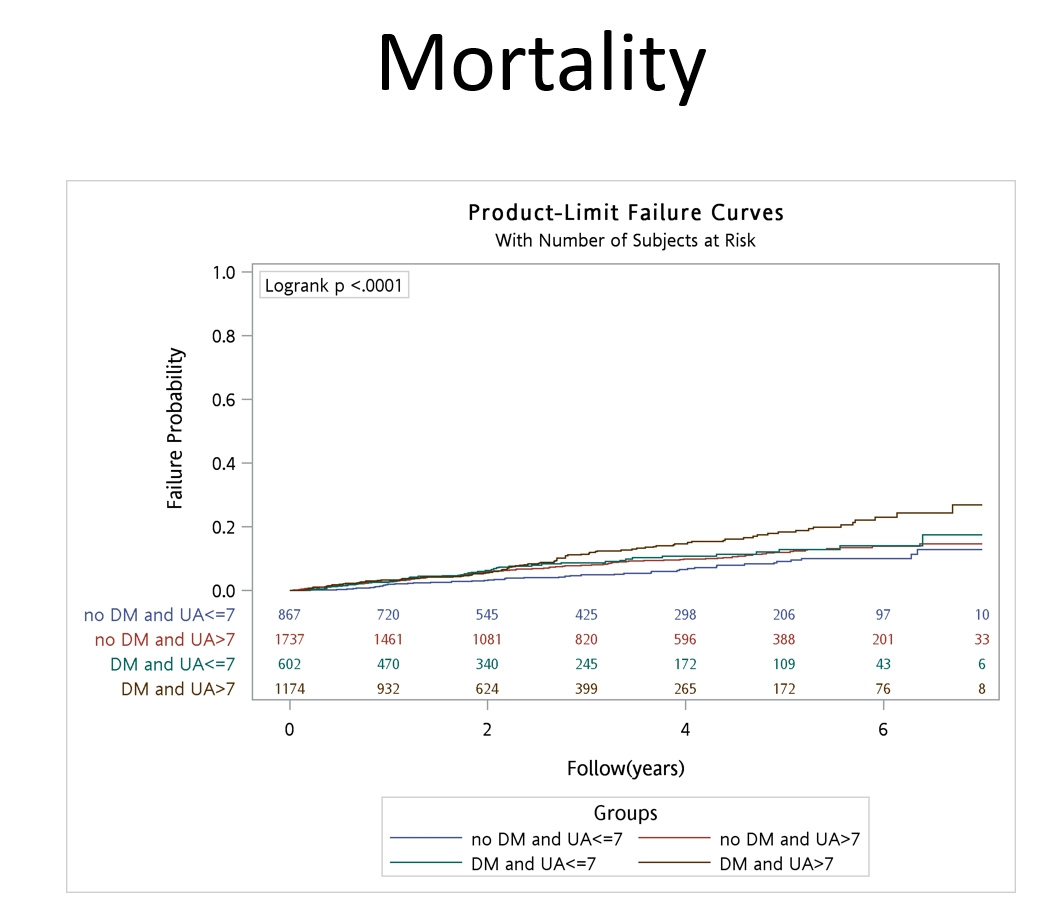

Supplement: Supplementary file 1 — Additional file 1. [file 12882_2022_2755_MOESM1_ESM.docx]
